# Antenatal Depression and its Associated Factors: Findings from Kuwait Birth Cohort Study

**DOI:** 10.1007/s44197-024-00223-7

**Published:** 2024-04-15

**Authors:** Reem Al-Sabah, Abdullah Al-Taiar, Ali H. Ziyab, Saeed Akhtar, Majeda S. Hammoud

**Affiliations:** 1 Department of Community Medicine and Behavioral Sciences, College of Medicine, Kuwait University, P.O. Box 24923, Safat, 13110 Kuwait; 2 School of Community and Environmental Health, College of Health Sciences, Old Dominion University, 3136 Health Sciences Building, 4608 Hampton Blvd, Norfolk, VA 23508 USA; 3 Department of Pediatrics, College of Medicine, Kuwait University, Safat, Kuwait

**Keywords:** Depression, Pregnancy, Edinburgh postnatal depression scale, Vitamin D, Folate, Vitamin B_12_

## Abstract

**Background:**

Pregnant and postpartum women are at high risk of depression due to hormonal and biological changes. Antenatal depression is understudied compared to postpartum depression and its predictors remain highly controversial.

**Aim:**

To estimate the prevalence of depressive symptoms during pregnancy and investigate factors associated with this condition including vitamin D, folate and Vitamin B_12_ among participants in the Kuwait Birth Study.

**Methods:**

Data collection occurred as part of the Kuwait Birth Cohort Study in which pregnant women were recruited in the second and third trimester during antenatal care visits. Data on antenatal depression were collected using the Edinburgh Postnatal Depression Scale (EPDS), considering a score of ≥ 13 as an indicator of depression. Logistic regression was used to investigate factors associated with depressive symptoms in pregnant women.

**Results:**

Of 1108 participants in the Kuwait Birth Cohort study, 1070(96.6%) completed the EPDS. The prevalence of depressive symptoms was 21.03%(95%CI:18.62–23.59%) and 17.85%(95%CI:15.60-20.28%) as indicated by an EPDS ≥ 13 and EPDS ≥ 14 respectively. In the multivariable analysis, passive smoking at home, experiencing stressful life events during pregnancy, and a lower level of vitamin B_12_ were identified as predisposing factors. Conversely, having desire for the pregnancy and consumption of fruits and vegetables were inversely associated with depressive symptoms.

**Conclusion:**

Approximately, one fifth of pregnant women had depressive symptoms indicating the need to implement screening program for depression in pregnant women, a measure not systematically implemented in Kuwait. Specifically, screening efforts should focus on pregnant women with unintended pregnancies, exposure to passive smoking at home, and recent stressful live events.

**Supplementary Information:**

The online version contains supplementary material available at 10.1007/s44197-024-00223-7.

## Introduction

Antenatal depression is a common health condition with between 10 and 20% of women experiencing depressive symptoms during pregnancy [1–4]. This is even higher among adolescent mothers [2, 5, 6] and those with a history of depression before becoming pregnant [7]. Depression during pregnancy is usually overlooked primarily because its symptoms can imitate those of normal pregnancy [8] and the fact that healthcare providers are usually focused on physical health aspects of pregnancy [9]. The substantial hormonal changes associated with pregnancy, particularly steroid hormones that influence hypothalamic pituitary adrenal and hypothalamic pituitary gonadal axes is linked to mood disorders, which may explain why women during pregnancy and the postpartum period are extremely vulnerable to depressive symptoms [10].

Studies have shown that antenatal depression has negative consequences on both mothers and offspring. During pregnancy, depression may increase the risk of preeclampsia [11], failure to seek optimal antenatal care [12], miscarriage or preterm birth [13, 14], failure to have optimal diet required for the mother and the fetus [12], risky behavior such as smoking or drug abuse [12], risk of suicide [15], delivery by elective cesarean section [16], risk of postpartum depression [17, 18], having difficulty bonding with the child [19, 20], decreased breast feeding [21], and prolonged sick leave [12, 16]. With respect to the impact of antenatal depression on the offspring, studies have shown that maternal depression during pregnancy may increase the risk of preterm birth [13, 21], low birth weight [13, 22], and poor cognitive function [23, 24]. Unfortunately, the treatment of depression during pregnancy may also carry some adverse effects as most antidepression medications cross the placenta [25, 26]. Adverse effects of antidepression treatment in offspring include prenatal antidepressant exposure syndrome [27], autism spectrum disorder [28, 29] and attention-deficit/hyperactivity disorder [29] although these are not confirmed and remained under rigorous debate [30, 31].

Compared to postpartum depression, antenatal depression has received much less attention [32]. Identifying modifiable risk factors for antenatal depression is a prerequisite for interventions that aim to reduce the burden on mothers and their children. However, the evidence remains inconclusive about the link between antenatal depression and several potential risk factors including the lack of physical activity [33], pre-pregnancy obesity [34], and nutritional factors [35, 36] indicating the need for high quality primary research. In high-income Arab countries in the Gulf region, there is a lack of data on the prevalence of antenatal depression as well as factors predisposing to this condition. The few studies that have estimated the prevalence of depressive symptoms during pregnancy in these countries showed heterogenous estimates ranging from 17 to 57.5% [37–41]. This study aimed to estimate the prevalence of depressive symptoms during pregnancy and investigate its association with several potential risk factors including the lack of physical activity, pre-pregnancy obesity, and dietary pattern as well as several nutritional biological markers such as vitamin D, folate, and Vitamin B_12_.

## Methods

### Study Design and Study Participants

Participants in this study were recruited through the Kuwait Birth Cohort Study in which pregnant women were recruited in the second or third trimester during antenatal care visits. Ethical approval of this study was granted by the research ethics committee at the Ministry of Health, Kuwait (Ref: project 173/2014; date: February 14th, 2017), as well as the Institutional Review Board at Old Dominion University (Ref: 1,517,949). Prior to enrollment, written informed consent was obtained from all study participants.

### Data Collection

#### Measuring Depression (the Dependent Variable)

Data on depressive symptoms were collected using the Edinburgh Postnatal Depression Scale (EPDS) [42], which has been validated in our setting [43]. The EPDS is used to screen for depression in women during pregnancy and postpartum period, but it is not considered a diagnostic tool. It contains 10 items, each is scored from 0 to 3, hence the total score is between 0 and 30. The higher the score, the greater the level of depression symptoms. Several cutoff points have been used to define depression including EPDS score ≥ 10, EPDS score ≥ 13 and EPDS score ≥ 14. In this study, we considered EPDS score ≥ 13 as indicative of depression. A recent validation study of the Arabic version of EPDS showed that this score has 87% sensitivity and 90% specificity [43].

#### Measuring Explanatory Variables

Socio-demographic and lifestyle data were obtained through face-to-face interviews with pregnant women, conducted by a trained data collector. The data collection form was created, and pilot tested on 30 pregnant women who were not part of the main study. Dietary data were collected through questions sourced from the Kuwait Nutritional Surveillance System and WHO stepwise approach to noncommunicable disease risk factors surveillance [44]. Data regarding physical activity were gathered using the pregnancy physical activity questionnaire [45], which has been previously validated in a similar context [46]. Participants self-reported their pre-pregnancy weight and height, while current weight and height measurements were taken in a standardized manner. Clinical data, including comorbidity data, was extracted from medical records by a healthcare professional.

### Laboratory Methods

Blood samples were collected and assessed in the laboratory of Al Sabah Maternity Hospital, where these laboratory investigations are routinely carried out with strict adherence to quality control standards. Vitamin B_12_, folate, and 25-Hydroxyvitamin D were examined using the Cobas e601 analyzer manufactured by Roche Diagnostics GmbH in Mannheim, Germany. Serum vitamin B_12_ was quantified using the Roche commercial kit (Catalog #7,212,771,190), whereas the total folate in hemolyzed whole blood was measured using the Roche commercial kit (Catalog # 7,559,992,190). Finally, 25-Hydroxyvitamin D levels were determined using the Roche commercial kit (Catalog # 9,038,078,190). Vitamin D status was classified as deficiency/insufficiency (25-Hydroxyvitamin D < 75 nmol/L) and sufficiency (25-Hydroxyvitamin D ≥ 75 nmol/L) using acceptable cut-off points [47].

### Statistical Methods

Data were entered in specially designed database using EpiData and were analyzed by STATA Release 17. The total score from EPDS was calculated by adding together the scores for each of the ten items. As per most studies in the region [37, 41, 48], those with EPDS score of ≥ 13 were considered as having depression. It has been suggested that this score has 87% sensitivity and 90% specificity in our setting [43]. We also recategorized the score as per other cutoff points and reported the results in the text as a sensitivity analysis. For example, we used EPDS score ≥ 14, which has been associated with greater certainty of depression [49] and evaluated the impact of this on our findings. We used logistic regression models to investigate factors associated with depressive symptoms calculating the crude and adjusted odds ratio and their 95% confidence intervals (CI). Factors that showed *p* < 0.05 were deemed to be statistically significant. In multivariable analysis, variables with *p* < 20% were grouped based on the stress process model framework [50], which has been used before in antenatal depression [51, 52] and postpartum depression [53]. The first group included stressors (listed in Tables (1), (2) and (4)) and the second group included mediators (listed in Tables (3) and (5)). Variables from these groups were modeled separately with models assessed using the Pearson χ^2^ goodness-of-fit test.

**Table 1:** Association between antenatal depressive symptoms^1^ and socio-demographic factors in univariable analysis

Characteristics	Total	Prevalence		Crude Odds Ratio [95% CI]		p
		n	(%)			
Nationality						
Kuwaiti	253	39	(15.42)		[Ref.]	0.012
Non-Kuwait	817	186	(22.77)	1.62	[1.11–2.36]	
Age (year)						
< 25 years	115	27	(23.48)		[Ref.]	0.026
25- 29.99 years	316	48	(15.19)	0.58	[0.34–0.99]	
30- 34.99 years	353	84	(23.80)	1.02	[0.62–1.67]	
35 + years	285	66	(23.16)	0.98	[0.59–1.64]	
Mother’s Education						
Elementary School or less	28	6	(21.43)	1.03	[0.41–2.59]	0.996
Secondary (high school)	242	51	(21.07)	1.01	[0.71–1.44]	
University & above	795	166	(20.88)		[Ref.]	
Father Education						
Elementary School or less	27	6	(22.22)	1.07	[0.42–2.69]	0.959
Secondary (high school)	260	53	(20.38)	0.96	[0.68–1.36]	
University & above	778	164	(21.08)		[Ref.]	
Father monthly Income (Kuwaiti Dinar)						
Less than 500	252	52	(20.63)		[Ref.]	0.672
500 to 1000	296	58	(19.59)	0.94	[0.62–1.42]	
More than 1000	160	33	(20.63)	1.00	[0.61–1.63]	
Prefer not to tell	346	81	(23.41)	1.17	[0.79–1.74]	
Mother employment						
Housewife	564	135	(23.94)		[Ref.]	0.051
Paid employment	479	85	(17.75)	0.68	[0.5–0.93]	
Others	23	5	(21.74)	0.88	[0.32–2.42]	
Mother’s monthly Income (Kuwaiti Dinar)						
No specific income	584	139	(23.80)		[Ref.]	0.123
Less than 500	152	25	(16.45)	0.63	[0.39–1.01]	
500 to 1000	142	24	(16.90)	0.65	[0.40–1.05]	
More than 1000	74	12	(16.22)	0.62	[0.32–1.18]	
Prefer not to tell	110	25	(22.73)	0.94	[0.58–1.53]	
Husband married to another women						
No	1041	214	(20.56)		[Ref.]	0.007
Yes	26	11	(42.31)	2.83	[1.28–6.26]	
Type of housing						
Rented apartment	837	187	(22.34)		[Ref.]	0.011
Rented house	33	4	(12.12)	0.48	[0.17–1.38]	
Owned apartment	27	10	(37.04)	2.04	[0.92–4.54]	
Owned house	168	24	(14.29)	0.58	[0.36–0.92]	

^1^Defined as Edinburgh Postnatal Depression Scale score ≥ 13. Number in some variables do not add up to 1070 because of few missing values

**Table 2:** Association between antenatal depressive symptoms^1^ and reproductive factors as well as active and passive smoking in univariable analysis

Characteristics	Total	Prevalence		Crude Odds Ratio [95% CI]		p
		n	(%)			
Age of menarche (year)						
8-	143	31	(21.68)		[Ref.]	0.995
12-	304	65	(21.38)	0.98	[0.61–1.59]	
13-	304	63	(20.72)	0.94	[0.58–1.53]	
14-	300	64	(21.33)	0.98	[0.60–1.59]	
Do not remember	42	8	(19.05)	--	--	
Age at first marriage						
Lower tertile < 22 years	319	71	(22.26)		[Ref.]	0.590
Second tertile > 22 to < 25 years	344	66	(19.19)	0.83	[0.57–1.21]	
Third tertile 25 years or more	404	87	(21.53)	0.96	[0.67–1.37]	
Age at first pregnancy						
Lower tertile < 23 years	306	68	(22.22)		[Ref.]	0.114
Second tertile > 23 to < 27 years	405	71	(17.53)	0.74	[0.51–1.08]	
Third tertile 27 years or more	352	82	(23.30)	1.06	[0.74–1.53]	
Number of male children						
Zero	521	106	(20.35)		[Ref.]	0.516
1 to 3 children	532	117	(21.99)	1.10	[0.82–1.48]	
4 or more children	17	2	(11.76)	0.52	[0.12–2.32]	
Number of female children						
Zero	524	102	(19.47)		[Ref.]	0.096
1 to 3 children	525	115	(21.90)	1.16	[0.86–1.56]	
4 or more children	21	8	(38.10)	2.55	[1.03–6.30]	
Time since the last delivery (months)						
≤ 18 months	104	22	(21.15)		[Ref.]	0.445
19 to 24 months	68	9	(13.24)	0.57	[0.24–1.32]	
25 month or above	553	120	(21.70)	1.03	[0.62–1.72]	
No previous pregnancy/delivery	345	74	(21.45)	1.02	[0.60–1.74]	
Wanted to get pregnant						
No	331	92	(27.79)		[Ref.]	< 0.001
Yes	733	132	(18.01)	0.57	[0.42–0.77]	
Became pregnant while using contraception						
No	982	200	(20.37)		[Ref.]	0.049
Yes	77	23	(29.87)	1.66	[0.99–2.78]	
Treatment to help get pregnant						
No	964	205	(21.27)		[Ref.]	0.732
Yes	101	20	(19.80)	0.91	[0.55–1.53]	
Ever had an abortion or miscarriage						
No	655	133	(20.31)		[Ref.]	0.466
Yes	415	92	(22.17)	1.12	[0.83–1.51]	
Smoking cigarettes/shisha during the last 9 months						
No	1,014	211	(20.81)		[Ref.]	0.454
Yes	56	14	(25.00)	1.27	[0.68–2.37]	
Passive smoking at home						
No	671	122	(18.18)		[Ref.]	0.003
Yes	399	103	(25.81)	1.56	[1.16–2.11]	

^1^Defined as Edinburgh Postnatal Depression Scale score ≥ 13. Number in some variables do not add up to 1070 because of few missing values

## Results

Of 1108 participants in Kuwait Birth Cohort study, 1070 (96.6%) completed EPDS. The mean (SD) age was 31.46 (5.28) years, and the majority (76.31%) were non-Kuwaiti. Most women were housewives (52.91%) and reported having no specific monthly income (54.99%). Other socio-demographic factors of the study participants can be gleaned from Table (1). The median (IQR) of EPDS score was 6 (11), while 14 (1.31%) pregnant women thought of harming themselves (chose responses other than “never” in item 10 in EPDS). Of those who completed the EPDS, 225 (21.03%; 95%CI: 18.62–23.59%) had depressive symptoms as defined by EPDS score ≥ 13, which was significantly higher in non-Kuwaiti compared to Kuwaiti mothers (22.77% vs. 15.42%; *p* = 0.012). The prevalence of depressive symptoms as define by EPDS score ≥ 14 was 17.85% (95%CI: 15.60-20.28%).

Table (1) shows the association between depressive symptoms and socio-demographic factors in univariable analysis. Demographic factors that were found to be associated with depressive symptoms included nationality (*p* = 0.012), age (*p* = 0.026), husband being married to another women (*p* = 0.007), and type of housing (*p* = 0.011). Similarly, women who wished to be pregnant were less likely to have depressive symptoms, crude odds ratio (COR) 0.57 (95%CI: 0.42–0.77); *p* < 0.001; while women who became pregnant while taking contraception were more likely to have depressive symptoms, COR 1.66 (95%CI: 0.99–2.78); *p* = 0.049 (Table 2). Additionally, women who were exposed to passive smoking at home were more likely to have depressive symptoms, COR 1.56 (95%CI: 1.16–2.11); *p* = 0.003 (Table 2). Of the dietary and lifestyle factors, taking supplements during pregnancy were inversely associated with depressive symptoms, COR 0.44 (95%CI: 0.25–0.76); *p* = 0.003; while consumption of fresh fruits or fresh vegetables were inversely associated with depressive symptoms (*p* < 0.001) (Table 3). Surprisingly, the total physical activity was positively associated with depressive symptoms (*p* < 0.001) Table (3). None of the comorbidities during pregnancy showed an association with depressive symptoms but having a stressful life event such as death of a relative or close friend was significantly associated with depressive symptoms, COR 2.61(95%CI: 1.86–3.67); *p* < 0.001 (Table 4). Apart from vitamin B_12_, which showed inverse association with depressive symptoms (*p* = 0.009), none of the laboratory factors including iron (*p* = 0.884), folate (*p* = 0.962) or vitamin D (*p* = 0.093) showed significant association with depressive symptoms (Table 5).

**Table 3:** Association between antenatal depressive symptoms^1^ and supplements and dietary factors as well as physical activity and pre-pregnancy BMI among in univariable analysis

Characteristics	Total	Prevalence		Crude Odds Ratio [95% CI]			p
		n	(%)				
Current use of supplements and vitamins							
No	60	22	(36.67)			[Ref.]	0.003
Yes	999	203	(20.32)	0.44		[0.25–0.76]	
Fasting during pregnancy							
No	491	99	(20.16)			[Ref.]	0.440
Yes	570	126	(22.11)	1.12		[0.83–1.51]	
Eating meat during the last 3 months							
I eat both meat and fish	821	164	(19.98)			[Ref.]	0.215
I avoid meat, but eat fish	69	26	(37.68)	2.42		[1.44–4.06]	
I avoid fish, but eat meat	103	19	(18.45)	0.90		[0.53–1.53]	
I avoid both fish and meat	49	11	(22.45)	1.16		[0.58–2.32]	
I am vegetarian	12	5	(41.67)	2.86		[0.89–9.13]	
Consumptions of carbonated drinks during the last 3 months							
Never or almost never	597	123	(20.60)			[Ref.]	0.696
1–2 glasses per week	262	59	(22.52)	1.12		[0.79–1.59]	
3–4 glasses per week	97	20	(20.62)	1.00		[0.59–1.70]	
5–6 glasses per week	37	6	(16.22)	0.74		[0.30–1.83]	
7 glasses per week or more	63	17	(26.98)	1.42		[0.79–2.57]	
Consumption of caned sugar-sweetened beverages							
Never or almost never	458	78	(17.03)			[Ref.]	0.733
1–2 glasses per week	307	55	(17.92)	0.87		[0.61–1.25]	
3–4 glasses per week	161	31	(19.25)	1.13		[0.74–1.73]	
5 glasses per week or more	127	24	(18.90)	0.99		[0.61–1.60]	
Consumption of fresh fruit juices							
Never or almost never	276	66	(23.91)			[Ref.]	0.516
1–2 glasses per week	388	76	(19.59)	0.77		[0.53–1.12]	
3–4 glasses per week	210	40	(19.05)	0.75		[0.48–1.16]	
5–6 glasses per week	86	19	(22.09)	0.90		[0.50–1.61]	
7 glasses per week or more	96	24	(25.00)	1.06		[0.62–1.82]	
Consumption of energy drinks							
Never or almost never	1,043	220	(21.09)			[Ref.]	0.129
One glasses per week or more	13	5	(38.46)	2.34		[0.76–7.22]	
Consumption of fresh fruits (days per week)							
≤ 2 days a week	263	89	(33.84)			[Ref.]	< 0.001
3–5 days a week	291	45	(15.46)	0.36		[0.24–0.54]	
≥ 6 days a week	495	88	(17.78)	0.42		[0.30–0.60]	
Consumption of fresh vegetables (days per week)							
≤ 2 days a week	309	100	(32.36)			[Ref.]	< 0.001
3–5 days a week	279	46	(16.49)	0.41		[0.28–0.61]	
≥ 6 days a week	452	76	(16.81)	0.42		[0.30–0.59]	
Physical activity (total MET)							
Low	354	52	(14.69)		[Ref.]		< 0.001
Middle	357	69	(19.33)	1.39	[0.93–2.06]		
High	355	103	(29.01)	2.37	[1.63–3.45]		
Pre-pregnancy BMI categories							
Underweight	24	3	(12.50)	0.65	[0.19–2.25]		0.207
Healthy weight	299	54	(18.06)		[Ref.]		
Overweight	363	87	(23.97)	1.43	[0.98–2.09]		
Obesity	276	60	(21.74)	1.26	[0.83–1.90]		

^1^Defined as Edinburgh Postnatal Depression Scale score ≥ 13. Number in some variables do not add up to 1070 because of few missing values

**Table 4:** Association between antenatal depressive symptoms^1^ and comorbidities, fasting during pregnancy, and stressful life event during pregnancy in univariable analysis

Characteristics	Total	Prevalence		Crude Odds Ratio [95% CI]		p	
		n	(%)				
Hypertension diagnosed							
No	985	202	(20.51)		[Ref.]	0.095	
Yes	77	22	(28.57)	1.55	[0.92–2.60]		
Gestational diabetes							
No	877	191	(21.78)		[Ref.]	0.184	
Yes	184	32	(17.39)	0.76	[0.50–1.14]		
Type 2 diabetes							
No	1,046	220	(21.03)		[Ref.]	0.699	
Yes	16	4	(25.00)	1.25	[0.40–3.92]		
Type 1 diabetes							
No	1,047	223	(21.30)		[Ref.]	0.168	
Yes	15	1	(6.67)	0.26	[0.03–2.02]		
Bleeding during the last 3 months							
No	959	202	(21.06)		[Ref.]	0.944	
Yes	103	22	(21.36)	1.02	[0.62–1.67]		
Other disease conditions							
No	876	185	(21.12)		[Ref.]	0.954	
Yes	183	39	(21.31)	1.01	[0.68–1.49]		
Using medications other than supplements							
No	736	158	(21.47)		[Ref.]	0.791	
Yes	323	67	(20.74)	0.96	[0.69–1.32]		
Stressful life event							
No	862	154	(17.87)		[Ref.]	< 0.001	
Yes	196	71	(36.22)	2.61	[1.86–3.67]		

^1^Defined as Edinburgh Postnatal Depression Scale score ≥ 13. Number in some variables do not add up to 1070 because of few missing values

**Table 5:** Association between antenatal depressive symptoms^1^ and laboratory factors in univariable analysis

Characteristics	Total	Prevalence		Crude Odds Ratio [95% CI]		p
		n	(%)			
Iron (umol/L)						
< 9 umol/L	325	71	(21.85)		[Ref.]	0.884
9–30 umol/L (normal)	708	146	(20.62)	0.93	[0.67–1.28]	
> 30 umol/L	31	7	(22.58)	1.04	[0.43–2.52]	
Ferritin ug/L						
< 13 ug/L	340	80	(23.53)		[Ref.]	0.337
13–150 ug/L (normal)	683	134	(19.62)	0.79	[0.58–1.08]	
> 150 ug/L	44	10	(22.73)	0.95	[0.45–2.02]	
Anemia						
No	768	159	(20.70)		[Ref.]	0.677
Yes	302	66	(21.85)	1.07	[0.77–1.48]	
Vitamin B_12_ (pg/L)						
≥ 180 pg/ml	729	134	(18.38)		[Ref.]	0.009
130–179 pg/ml	225	60	(26.67)	1.61	[1.14–2.29]	
< 130 pg/ml	113	30	(26.55)	1.60	[1.01–2.54]	
RBC Folate ng/mL						
< 263 ng/mL	113	24	(21.24)		[Ref.]	0.962
≥ 263 ng/mL	955	201	(21.05)	0.99	[0.61–1.59]	
Calcium						
> 5.52 mmol/L	9	2	(22.22)	1.18	[0.24–5.82]	0.651
2.2–5.52 mmol/L	365	71	(19.45)		[Ref.]	
< 2.2 mmol/L	690	151	(21.88)	1.16	[0.84–1.59]	
Vitamin D status						
Deficiency/insufficiency (25OHD < 75 nmol/L)	817	181	(22.15)		[Ref.]	0.093
Sufficiency (25OHD ≥ 75 nmol/L)	250	43	(17.20)	0.73	[0.50–1.05]	

^1^Defined as Edinburgh Postnatal Depression Scale score ≥ 13. 25OHD: 25-Hydroxyvitamin D. Number in some variables do not add up to 1070 because of few missing values

**Table 6:** Factors associated with antenatal depressive symptoms^1^ in Kuwait birth cohort study in adjusted model

Characteristics	Total	Prevalence		Adjusted Odds Ratio [95% CI]		p
		n	(%)			
Group (1): Stressors^2^						
Wanted to get pregnant						
No	331	92	(27.79)		[Ref.]	0.028
Yes	733	132	(18.01)	0.67	[0.47–0.96]	
Passive smoking at home						
No	671	122	(18.18)		[Ref.]	0.011
Yes	399	103	(25.81)	1.52	[1.10–2.10]	
Stressful life event						
No	862	154	(17.87)		[Ref.]	< 0.001
Yes	196	71	(36.22)	2.20	[1.53–3.17]	
Group (2): Mediators^3^						
Consumption of fresh fruits (days per week)						
≤ 2 days a week	263	89	(33.84)		[Ref.]	0.021
3–5 days a week	291	45	(15.46)	0.54	[0.34–0.86]	
≥ 6 days a week	495	88	(17.78)	0.61	[0.40–0.93]	
Consumption of fresh vegetables (days per week)						
≤ 2 days a week	309	100	(32.36)		[Ref.]	0.023
3–5 days a week	279	46	(16.49)	0.57	[0.36–0.90]	
≥ 6 days a week	452	76	(16.81)	0.60	[0.39–0.91]	
Physical activity (total MET)						
Low	354	52	(14.69)		[Ref.]	0.001
Middle	357	69	(19.33)	1.32	[0.87-2.00]	
High	355	103	(29.01)	2.04	[1.38–3.02]	
Vitamin B_12_ (pg/L)						
≥ 180 pg/ml	729	134	(18.38)		[Ref.]	0.038
130–179 pg/ml	225	60	(26.67)	1.54	[1.06–2.23]	
< 130 pg/ml	113	30	(26.55)	1.52	[0.93–2.48]	

^1^Defined as Edinburgh Postnatal Depression Scale score ≥ 13

^2^Adjusted for nationality (*p* = 0.093), age group (*p* = 0.170), women employment (*p* = 0.912), mother income (*p* = 0.473), number of female children (*p* = 0.387), husband currently married to another women (*p* = 0.060), type of housing (*p* = 0.128), age at first pregnancy (categorized)(*p* = 0.328), becoming pregnant while using contraception (*p* = 0.475), presence of hypertension (*p* = 0.538), and gestational diabetes (*p* = 0.188)

^3^Adjusted for using supplement (*p* = 0.060), consumption of energy drinks (*p* = 0.650), and vitamin D (*p* = 0.387)

In multivariable analysis, out of the stressors’ group, the desire to get pregnant was negatively associated with depressive symptoms, adjusted odds ratio (AOR) 0.67(95%CI: 0.47–0.96); *p* = 0.028 (Table 6). Both passive smoking at home and stressful life events during pregnancy were positively associated with depressive symptoms, AOR 1.52(95%CI: 1.10–2.10); *p* = 0.011 and 2.20 (95%CI: 1.53–3.17); *p* < 0.001, respectively. Weekly consumption of each fresh fruits and fresh vegetables were inversely associated with depressive symptoms; *p* = 0.021 and *p* = 0.023, respectively. Surprisingly, physical activity was positively associated with depressive symptoms with those in the highest tertile being more likely to report symptoms of depression compared to those in the lowest tertile, AOR 2.04(95%CI: 1.38–3.02); *p* = 0.001. Finally, vitamin B_12_ was inversely associated with depressive symptoms, *p* = 0.038 (Table 6). The analysis above was repeated with the depressive symptoms defined as EPDS score ≥ 14, which show some differences in the multivariable model but minimal changes in our conclusions (See supplementary Tables 1–6). For example, passive smoking and fruit consumption lost statistical significance, (*p* = 0.087) and (*p* = 0.070), respectively. Also, vitamin B_12_ was no longer associated with depressive symptoms (*p* = 0.407) and instead current consumption of supplements (*p* = 0.001), and meat consumption (*p* = 0.004) were significantly associated with depressive symptoms. Finally, pre-pregnancy Body Mass Index (BMI) was significantly associated with antenatal depressive symptoms with women who were overweight being more likely to have depressive symptoms (*p* = 0.039).

## Discussion

This study aimed to estimate the prevalence of depressive symptoms during pregnancy in Kuwait and investigated factors associated with these symptoms during pregnancy. In particular, we aimed to investigate the association between depressive symptoms during pregnancy and socio-demographic, reproductive, and clinical factors as well as dietary pattern and biological nutritional markers such as vitamin D, folate and vitamin B_12_. We found that around 21% of pregnant women had depressive symptoms as per the EPDS score ≥ 13 while 18% of pregnant women had depressive symptoms defined as an EPDS score ≥ 14. Several factors were found to be positively associated with depressive symptoms during pregnancy including having stressful life events, and passive smoking at home as well as lower levels of vitamin B_12_. Factors that were found to be negatively associated with depressive symptoms during pregnancy included planned pregnancy and consumptions of fresh fruits and fresh vegetables.

The prevalence of depressive symptoms during pregnancy was found to be 21% (EPDS score ≥ 13) that became 18% with an EPDS score ≥ 14, which is similar to that reported from Oman (24%) [37]. However, it is lower than the estimates reported from Saudi Arabia, where small studies showed that between 32% [48] and 57.5% [41] of pregnant women had depressive symptoms as well as other low-income countries in the region such as Egypt (63%) [54] and Jordan (57%) [55]. It is also lower than that reported from low- or middle-income countries such as China (28.5%) [32], Ethiopia (25%) [56], Tanzania (33.8%) [57], Brazil (35.6%) [58], and Iran (37%) [59]. An earlier study in Kuwait estimated the prevalence of depressive symptoms during pregnancy to be 20% based on a lower cutoff point of EPDS score ≥ 10 to define depression [39]. Using this cutoff point in our study would yield a prevalence of 31.87%. The difference between our estimate and earlier estimate could be due to the fact that we recruited women from a hospital, while the earlier study recruited their participants from the general population. Our findings highlight the need to implement systematic screening for depression during pregnancy in Kuwait, as per the recommendation by the American College of Obstetricians and Gynecologists as well as the US preventive services taskforce (USPSTF) [60, 61]. It is important that pregnant women with positive screening are evaluated further for diagnosis and, if appropriate, are referred for evidence-based mental healthcare services as screening per say would not provide benefit beyond self-awareness.

In univariable analysis, several factors in the first conceptual domain of stress process model [50] were found to be associated with antenatal depression. Being non-Kuwaiti was positively associated with depressive symptoms which may be explained by the fact that they are away from their extended families in their own countries in addition to the advantages that Kuwaiti citizens enjoy compared to non-Kuwaiti such as free healthcare, education, housing, as well as subsidized food items. Of note is the positive association between having a husband married to another women and depressive symptoms, which is another major stressor. Although only a small number of women reported a husband married to another women, this particular group of women need to be screened for antenatal depression. Unlike an earlier study in Saudi Arabia [41], which showed association between depressive symptoms in pregnant women and the number of children or the number of daughters, we found no association between depressive symptoms during pregnancy and these factors in univariable or multivariable analysis.

In multivariable analysis, women who wanted to get pregnant were less likely to have depressive symptoms, which is similar to that reported from other studies that showed unplanned pregnancy [56, 62] or unintended pregnancy [63] as a significant predictor for antenatal depression. Having a stressful life event during pregnancy was significantly associated with depressive symptoms in our study, which is similar to the conclusion of a systematic literature review that showed stressful live events as a significant predictor for depressive symptoms in pregnant women [63]. Similarly, passive smoking at home was positively associated with depressive symptoms in our setting, which is similar to that reported from other settings [48, 64]. A recent literature review on this issue, concluded that secondhand smoking is associated with a significant increase in the odds of antenatal depression [65]. These findings are highly valuable to help identify pregnant women at risk for antenatal depression in our setting.

It was surprising to find that pregnant women who are at the highest tertile of physical activity are more likely to have depressive symptoms compared to those in the lowest tertile as the opposite was expected. A recent literature review of 11 studies on the association of physical activity during pregnancy and postpartum depression highlighted the heterogeneous results with one study showing positive association-like ours- and nine studies showing no association, although the authors concluded that physical activity may prevent postpartum depression [33]. Another literature review of the trials on yoga-based exercises or other defined exercises during pregnancy concluded that physical activity during pregnancy may reduce depressive symptoms in the perinatal period [66]. It should be clear that most of the physical activity in our setting was reported in the domain of household work rather than regular physical exercises or yoga-based exercise. The higher level of physical activity among our participants reflects more repetitive house chore (e.g., taking care of children) as outlined in the physical activity questionnaire, which may be an added burden that may predispose women to antenatal depression.

We found no evidence for the association between pre-pregnancy self-reported BMI and depressive symptoms during pregnancy. Studies that investigated the association between pre-pregnancy BMI and antenatal or postnatal depression have shown controversial findings (reviewed recently by Dachew et al. [34]). Although most reviews concluded that obesity before pregnancy is a significant risk factor for antenatal depression despite the high degree of conflicting results from individual studies [34, 67], there is strong believe that this association appeared because of unmeasured cofounders or residual confounding [34]. It has been suggested that the lack of association between pre-pregnancy obesity and antenatal depression could be explained by the fact that women during pregnancy may view high body weight as a positive sign of normal pregnancy [68, 69]. Women are more likely to think this way in the second or third trimester, which may explain our findings. Unfortunately, none of the reviews stratified the analysis by trimester despite the fact that the association may become weaker during the second or third trimester. Nevertheless, pre-pregnancy BMI was significantly associated with depressive symptoms when we used EPDS score ≥ 14 as a cutoff point (Supplementary Table 6).

The role of nutrition in developing depression in general and during pregnancy has attracted a lot of attention. The theoretical plausibility that can explain how several nutrients may affect synthesis or regulation of some neurotransmitters hence regulate mood have been presented in the literature [70–72]. This created strong motivation to demonstrate the impact of nutrition on depression using epidemiological studies, particularly during pregnancy, a period in which nutritional requirements increases significantly. Overall, the evidence for the link between dietary patterns and depression is limited and inconsistent [35, 36]. In our study, we collected data on dietary pattern and measured multiple biological nutritional markers including iron, folate, vitamin D, and vitamin B_12_. We found consumption of fresh fruits and vegetables as a significant predictor for depressive symptoms in pregnant women in univariable and multivariable analyses (Tables 3 and 6). In a recent small trial, a higher consumption of fruits showed an inverse association with postpartum depression [73]. Also, a brief intervention with diet that includes fruits and vegetable has been shown to reduce depressive symptoms in adults [74]. An earlier literature review showed that dietary pattern characterized by a high intake of fruit, vegetables, whole grain, fish, olive oil, low-fat dairy, and antioxidants; and low intakes of animal foods was associated with a decreased risk of depression in general adults [36]. Consumption of fruits and vegetables provides a wide range of nutrients, which may explain this association in our study. As we collected dietary data and measured antenatal depression at the same time, it is possible that the association may reflect a reverse causality (women with depression became less likely to take care of their nutrition hence consume less fruits and vegetables compared to women without depression).

One of the major strengths of our study is measuring multiple biological nutritional biomarkers such as vitamin B_12_, vitamin D and folate. As mentioned above, we found no association between folate and depressive symptoms during pregnancy in univariable or multivariable analysis, which is consistent with a recent review that concluded that there is no association between folic acid intervention and EDPS score during and post pregnancy [75]. Our finding showed that low vitamin B_12_ levels are associated with depressive symptoms in univariable and multivariable analysis (Tables 5 and 6). Despite the biological plausibility that can explain the impact of vitamin B_12_ on mental health, epidemiological studies do not generally support the link between vitamin B_12_ and antenatal depression. An analysis of National Health and Nutrition Examination Survey (NHANES) data showed that vitamin B_12_ levels were associated with antenatal depression in univariable and multivariable analysis although depression was measured using the Patient Health Questionnaire-9 [76]. In the birth cohort of Singapore (*n* = 709), plasma vitamin B_12_ concentrations were not associated with perinatal depression [77]. A recent literature review showed that only 5 studies addressed this issue and that there was no association between vitamin B_12_ deficiency or insufficiency and depression during pregnancy [78]. Finally, a recent literature review of 11 randomized control trials (six trials on vitamin B_12_ alone and five for vitamin B complex) concluded that vitamin B_12_ supplementation is ineffective in improving depressive symptoms in patients without advanced neurological disorder [79]. It is worth noting that we found no association between vitamin B_12_ and depressive symptoms when we used EPDS score ≥ 14 as a cutoff point (Supplementary Table 6).

Vitamin D was not significantly associated with depressive symptoms during pregnancy in our study. Despite the biological plausibility that can explain the link between vitamin D and antenatal depression, a recent literature review on this topic showed that the evidence is poor and inconclusive [80]. In fact, multiple reviews over the past decade have consistently called for better quality of research on this topic [80–83]. One of the major weaknesses of observational studies on this issue is the lack of adjusting the association between vitamin D and antenatal depression for season in which blood samples were taken. We adjusted for the season in which blood sample was taken and there was no change in our findings, AOR 0.76(95%CI: 0.52–1.10) (*p* = 0.141). This analysis, however, showed that women recruited in Summer or Spring had higher prevalence of depressive symptoms compared to those recruited in Fall or Winter (*p* = 0.013).

This study has several strengths including the large sample size and utilization of EPDS, a validated tool that has been widely used to measure depressive symptoms in numerous studies. Nonetheless, it is essential to recognize that EPDS is merely a screening tool for depression in pregnant and postpartum women, and by no mean can be considered sufficient for the diagnosis of depression, which requires clinical assessment. The other limitation of this study is the lack of data on social support. Also, despite the fact that our study was birth cohort, this analysis was based on data collected at baseline hence the data on the risk factors and antenatal depression were collected at that same time, which makes us less certain about the temporal relationship between some risk factors and depressive symptoms. For example, pregnant women with depression may neglect their diet hence eat less fruits and vegetable compared to women without depression, which makes it difficult to assume that the association between diet and depressive symptoms is causal.

## Conclusion

In conclusion, about one fifth of pregnant women had depressive symptoms based on the EPDS, which suggest the need to implement screening program for depression among pregnant women in Kuwait. Despite the well-accepted recommendation of screening for depression during pregnancy [60, 61], this is not systematically implemented in Kuwait. Pregnant women with positive screening should be evaluated for diagnosis and, if appropriate, are referred for evidence-based mental healthcare services. Pregnant women with unintended pregnancy, passive smoking at home, and stressful life events should be targeted by the screening program for antenatal depression. Finally, further studies are recommended to elucidate the impact of lifestyle factors such as physical activity and dietary pattern on the risk of antenatal depression.

## Electronic Supplementary Material

Below is the link to the electronic supplementary material.

Supplementary Material 1

## Data Availability

Data analyzed in this study will be available from the corresponding authors upon reasonable request.

## Abbreviations

EPDS: Edinburgh Postnatal Depression Scale
CI: Confidence Interval
SD: Standard Deviation
IQR: Interquartile Range
COR: Crude Odds Ratio
AOR: Adjusted Odds Ratio
BMI: Body Mass Index
