# Supplementary material for: Antenatal Depression and its Associated Factors: Findings from Kuwait Birth Cohort Study
Source: J Epidemiol Glob Health. 2024 Apr 15;14(3):847–59. doi: 10.1007/s44197-024-00223-7 (PMC11442740; doi:10.1007/s44197-024-00223-7)
Supplement: Supplementary file 1 — Supplementary Material 1 [file 44197_2024_223_MOESM1_ESM.docx]

**Table (1).** Association between antenatal depressive symptoms (defined as EPDS^1^ score≥ 14) and socio-demographic factors in univariable analysis.

| Characteristics | Total | Prevalence | | Crude Odds Ratio [95% CI] | | p |
| --- | --- | --- | --- | --- | --- | --- |
|  |  | n | (%) |  |  |  |
| Nationality |  |  |  |  |  |  |
| Kuwaiti | 253 | 32 | (12.65) |  | [Ref.] | 0.014 |
| Non-Kuwait | 817 | 159 | (19.46) | 1.67 | [1.11-2.51] |  |
| Age (year) |  |  |  |  |  |  |
| < 25 years | 115 | 21 | (18.26) |  | [Ref.] | 0.078 |
| 25- 29.99 years | 316 | 42 | (13.29) | 0.69 | [0.39- 1.22] |  |
| 30- 34.99 years | 353 | 73 | (20.68) | 1.17 | [0.68-2.00] |  |
| 35+ years | 285 | 55 | (19.30) | 1.07 | [0.61-1.87] |  |
| Mother’s Education |  |  |  |  |  |  |
| Elementary School or less | 28 | 6 | (21.43) | 1.26 | [0.50-3.18] | 0.881 |
| Secondary (high school) | 242 | 43 | (17.77) | 1.00 | [0.69-1.46] |  |
| University & above | 795 | 141 | (17.74) |  | [Ref.] |  |
| Father Education |  |  |  |  |  |  |
| Elementary School or less | 27 | 5 | (18.52) | 1.04 | [0.38-2.78] | 0.921 |
| Secondary (high school) | 260 | 44 | (16.92) | 0.93 | [0.64-1.35] |  |
| University & above | 778 | 140 | (17.99) |  | [Ref.] |  |
| Father monthly Income (Kuwaiti Dinar) |  |  |  |  |  |  |
| Less than 500 | 252 | 52 | (20.63) |  | [Ref.] | 0.672 |
| 500 to 1000 | 296 | 58 | (19.59) | 0.94 | [0.62–1.42] |  |
| More than 1000 | 160 | 33 | (20.63) | 1.00 | [0.61-1.63] |  |
| Prefer not to tell | 346 | 81 | (23.41) | 1.17 | [0.79 -1.74] |  |
| Mother employment |  |  |  |  |  |  |
| Housewife | 564 | 114 | (20.21) |  | [Ref.] | 0.112 |
| Paid employment | 479 | 74 | (15.45) | 0.72 | [0.52-0.99] |  |
| Others | 23 | 3 | (13.04) | 0.59 | [0.17-2.03] |  |
| Mother’s monthly Income (Kuwaiti Dinar) |  |  |  |  |  |  |
| No specific income | 584 | 116 | (19.86) |  | [Ref.] | 0.216 |
| Less than 500 | 152 | 21 | (13.82) | 0.65 | [0.39–1.07] |  |
| 500 to 1000 | 142 | 20 | (14.08) | 0.66 | [0.39–1.11] |  |
| More than 1000 | 74 | 11 | (14.86) | 0.70 | [0.36-1.38] |  |
| Prefer not to tell | 110 | 23 | (20.91) | 1.07 | [0.64-1.76] |  |
| Husband married to another women |  |  |  |  |  |  |
| No | 1041 | 181 | (17.39) |  | [Ref.] | 0.006 |
| Yes | 26 | 10 | (38.46) | 2.97 | [1.33-6.65] |  |
| Type of housing |  |  |  |  |  |  |
| Rented apartment | 837 | 158 | (18.88) |  | [Ref.] | 0.011 |
| Rented house | 33 | 3 | (9.09) | 0.43 | [0.13-1.42] |  |
| Owned apartment | 27 | 7 | (25.93) | 1.50 | [0.62-3.62] |  |
| Owned house | 168 | 23 | (13.69) | 0.68 | [0.42-1.09] |  |
| ^1^ Edinburgh Postnatal Depression Scale. Number in some variables do not add up to 1070 because of few missing values. | | | | | | |

**Table (2).** Association between antenatal depressive symptoms (defined as EPDS^1^ score≥ 14) and reproductive factors as well as active and passive smoking in univariable analysis.

| Characteristics | Total | Prevalence | | Crude Odds Ratio [95% CI] | | p |
| --- | --- | --- | --- | --- | --- | --- |
|  |  | n | (%) |  |  |  |
| Age of menarche (year) |  |  |  |  |  |  |
| 8- | 143 | 24 | (16.78) |  | [Ref.] | 0.970 |
| 12- | 304 | 54 | (17.76) | 1.07 | [0.63-1.82] |  |
| 13- | 304 | 55 | (18.09) | 1.09 | [0.65-1.85] |  |
| 14- | 300 | 56 | (18.67) | 1.14 | [0.67-1.92] |  |
| Do not remember | 22 | 2 | (9.09) | -- | -- |  |
| Age at first marriage |  |  |  |  |  |  |
| Lower tertile <22 years | 319 | 58 | (18.18) |  | [Ref.] | 0.719 |
| Second tertile > 22 to < 25 years | 344 | 57 | (16.57) | 0.89 | [0.60-1.34] |  |
| Third tertile 25 years or more | 404 | 76 | (18.81) | 1.04 | [0.71-1.52] |  |
| Age at first pregnancy |  |  |  |  |  |  |
| Lower tertile <23 years | 306 | 54 | (17.65) |  | [Ref.] | 0.102 |
| Second tertile > 23 to < 27 years | 405 | 60 | (14.81) | 0.81 | [0.54-1.21] |  |
| Third tertile 27 years or more | 352 | 73 | (20.74) | 1.22 | [0.82-1.80] |  |
| Number of male children |  |  |  |  |  |  |
| Zero | 521 | 96 | (18.43) |  | [Ref.] | 0.742 |
| 1 to 3 children | 532 | 93 | (17.48) | 0.95 | [0.68-1.28] |  |
| 4 or more children | 17 | 2 | (11.76) | 0.59 | [0.13-2.62] |  |
| Number of female children |  |  |  |  |  |  |
| Zero | 524 | 91 | (17.37) |  | [Ref.] | 0.421 |
| 1 to 3 children | 525 | 94 | (17.90) | 1.04 | [0.75-1.42] |  |
| 4 or more children | 21 | 6 | (28.57) | 1.90 | [0.72-5.04] |  |
| Time since the last delivery (months) |  |  |  |  |  |  |
| ≤ 18 months | 104 | 19 | (18.27) |  | [Ref.] | 0.452 |
| 19 to 24 months | 68 | 8 | (11.76) | 0.60 | [0.24-1.45] |  |
| 25 month or above | 553 | 96 | (17.36) | 0.94 | [0.54-1.62] |  |
| No previous pregnancy/delivery | 345 | 68 | (19.71) | 1.10 | [0.62-1.93] |  |
| Wanted to get pregnant |  |  |  |  |  |  |
| No | 331 | 76 | (22.96) |  | [Ref.] | 0.004 |
| Yes | 733 | 115 | (15.69) | 0.62 | [0.45-0.86] |  |
| Became pregnant while using contraception |  |  |  |  |  |  |
| No | 982 | 174 | (17.72) |  | [Ref.] | 0.500 |
| Yes | 77 | 16 | (20.78) | 1.22 | [0.68-2.16] |  |
| Treatment to help get pregnant |  |  |  |  |  |  |
| No | 964 | 172 | (17.84) |  | [Ref.] | 0.809 |
| Yes | 101 | 19 | (18.81) | 1.07 | [0.63-1.80] |  |
| Ever had an abortion or miscarriage |  |  |  |  |  |  |
| No | 655 | 113 | (17.25) |  | [Ref.] | 0.521 |
| Yes | 415 | 78 | (18.80) | 1.11 | [0.81-1.53] |  |
| Smoking cigarettes/shisha during the last 9 months |  |  |  |  |  |  |
| No | 1,014 | 179 | (17.65) |  | [Ref.] | 0.473 |
| Yes | 56 | 12 | (21.43) | 1.27 | [0.66-2.46] |  |
| Passive smoking at home |  |  |  |  |  |  |
| No | 671 | 108 | (16.10) |  | [Ref.] | 0.052 |
| Yes | 399 | 83 | (20.80) | 1.37 | [1.00-1.88] |  |
| ^1^ Edinburgh Postnatal Depression Scale. Number in some variables do not add up to 1070 because of few missing values. | | | | | | |

**Table (3).** Association between antenatal depressive symptoms (defined as EPDS^1^ score≥ 14) and supplements and dietary factors as well as physical activity and pre-pregnancy BMI among in univariable analysis.

| Characteristics | Total | Prevalence | | Crude Odds Ratio [95% CI] | | | p |
| --- | --- | --- | --- | --- | --- | --- | --- |
|  |  | n | (%) |  | | |  |
| Current use of supplements and vitamins |  |  |  |  | |  |  |
| No | 60 | 22 | (36.67) |  | | [Ref.] | <0.001 |
| Yes | 999 | 169 | (16.92) | 0.35 | | [0.20-0.61] |  |
| Eating meat during the last 3 months |  |  |  |  | |  |  |
| I eat both meat and fish | 821 | 138 | (16.81) |  | | [Ref.] | 0.006 |
| I avoid meat, but eat fish | 69 | 22 | (31.88) | 2.31 | | [1.35-3.97] |  |
| I avoid fish, but eat meat | 103 | 17 | (16.50) | 0.98 | | [0.56-1.70] |  |
| I avoid both fish and meat | 49 | 9 | (18.37) | 1.11 | | [0.53-2.34] |  |
| I am vegetarian | 12 | 5 | (41.67) | 3.53 | | [1.10-11.30] |  |
| Consumptions of carbonated drinks during the last 3 months |  |  |  |  | |  |  |
| Never or almost never | 597 | 106 | (17.76) |  | | [Ref.] | 0.740 |
| 1-2 glasses per week | 262 | 47 | (17.94) | 1.01 | | [0.69-1.48] |  |
| 3-4 glasses per week | 97 | 18 | (18.56) | 1.05 | | [0.60-1.83] |  |
| 5 - 6 glasses per week | 37 | 5 | (13.51) | 0.72 | | [0.27- 1.90] |  |
| 7 glasses per week or more | 63 | 15 | (23.81) | 1.45 | | [0.78-2.68] |  |
| Consumption of caned sugar-sweetened beverages |  |  |  |  | |  |  |
| Never or almost never | 458 | 78 | (17.03) |  | | [Ref.] | 0.914 |
| 1-2 glasses per week | 307 | 55 | (17.92) | 1.06 | | [0.73-1.55] |  |
| 3-4 glasses per week | 161 | 31 | (19.25) | 1.16 | | [0.73-1.84] |  |
| 5 glasses per week or more | 127 | 27 | (21.26) | 1.13 | | [0.68-1.88] |  |
| Consumption of fresh fruit juices |  |  |  |  | |  |  |
| Never or almost never | 276 | 59 | (21.38) |  | | [Ref.] | 0.351 |
| 1-2 glasses per week | 388 | 67 | (17.27) | 0.77 | | [0.52-1.13] |  |
| 3-4 glasses per week | 210 | 30 | (14.29) | 0.61 | | [0.38-0.99] |  |
| 5 - 6 glasses per week | 86 | 17 | (19.77) | 0.91 | | [0.49-1.66] |  |
| 7 glasses per week or more | 96 | 18 | (18.75) | 0.85 | | [0.62-1.82] |  |
| Consumption of energy drinks |  |  |  |  | |  |  |
| Never or almost never | 1,043 | 188 | (18.02) |  | | [Ref.] | 0.638 |
| One glasses per week or more | 13 | 3 | (23.08) | 2.34 | | [0.76-7.22] |  |
| Consumption of fresh fruits (days per week) |  |  |  |  | |  |  |
| ≤ 2 days a week | 263 | 77 | (29.28) |  | | [Ref.] | <0.001 |
| 3-5 days a week | 291 | 40 | (13.75) | 0.38 | | [0.25-0.59] |  |
| ≥6 days a week | 495 | 72 | (14.55) | 0.41 | | [0.28-0.59] |  |
| Consumption of fresh vegetables (days per week) |  |  |  |  | |  |  |
| ≤ 2 days a week | 309 | 87 | (28.16) |  | | [Ref.] | <0.001 |
| 3-5 days a week | 279 | 38 | (13.62) | 0.40 | | [0.26-0.61] |  |
| ≥6 days a week | 452 | 64 | (14.16) | 0.42 | | [0.29-0.60] |  |
| Physical activity (total MET) |  |  |  |  |  | |  |
| Low | 354 | 44 | (12.43) |  | [Ref.] | | <0.001 |
| Middle | 357 | 56 | (15.69) | 1.31 | [0.86-2.00] | |  |
| High | 355 | 90 | (25.35) | 2.39 | [1.61-3.55] | |  |
| Pre-pregnancy BMI categories |  |  |  |  |  | |  |
| Underweight | 24 | 3 | (12.50) | 0.65 | [0.19-2.25] | | 0.128 |
| Healthy weight | 299 | 43 | (14.38) |  | [Ref.] | |  |
| Overweight | 363 | 77 | (21.21) | 1.43 | [0.98-2.09] | |  |
| Obesity | 276 | 50 | (18.12) | 1.26 | [0.83-1.90] | |  |
| ^1^ Edinburgh Postnatal Depression Scale. Number in some variables do not add up to 1070 because of few missing values. | | | | | | | |

**Table (4).** Association between antenatal depressive symptoms (defined as EPDS^1^ score≥ 14) and comorbidities, fasting during pregnancy, and stressful life event during pregnancy in univariable analysis.

| Characteristics | Total | Prevalence | | Crude Odds Ratio [95% CI] | | p |
| --- | --- | --- | --- | --- | --- | --- |
|  |  | n | (%) |  |  |  |
| Hypertension diagnosed |  |  |  |  |  |  |
| No | 985 | 170 | (17.26) |  | [Ref.] | 0.055 |
| Yes | 77 | 20 | (25.97) | 1.68 | [0.98-2.87] |  |
| Gestational diabetes |  |  |  |  |  |  |
| No | 877 | 163 | (18.59) |  | [Ref.] | 0.151 |
| Yes | 184 | 26 | (14.13) | 0.72 | [0.46-1.13] |  |
| Type 2 diabetes |  |  |  |  |  |  |
| No | 1,046 | 187 | (17.88) |  | [Ref.] | 0.928 |
| Yes | 16 | 3 | (18.75) | 1.06 | [0.30-3.76] |  |
| Type 1 diabetes |  |  |  |  |  |  |
| No | 1,047 | 190 | (18.15) |  | [Ref.] | 0.069 |
| Yes | 15 | 0 | (0.00) | -- | [----] |  |
| Bleeding during the last 3 months |  |  |  |  |  |  |
| No | 959 | 170 | (0.671) |  | [Ref.] | 0.671 |
| Yes | 103 | 20 | (19.42) | 1.12 | [0.67-1.87] |  |
| Other disease condition |  |  |  |  |  |  |
| No | 876 | 157 | (17.92) |  | [Ref.] | 0.972 |
| Yes | 183 | 33 | (18.03) | 1.01 | [0.66-1.52] |  |
| Using medications other than supplements |  |  |  |  |  |  |
| No | 736 | 138 | (18.75) |  | [Ref.] | 0.362 |
| Yes | 323 | 53 | (16.41) | 0.85 | [0.60-1.20] |  |
| Fasting during pregnancy |  |  |  |  |  |  |
| No | 491 | 90 | (18.33) |  | [Ref.] | 0.796 |
| Yes | 570 | 101 | (17.72) | 0.96 | [0.70-1.31] |  |
| Stressful life event |  |  |  |  |  |  |
| No | 862 | 136 | (15.78) |  | [Ref.] | <0.001 |
| Yes | 196 | 55 | (28.06) | 2.08 | [1.45-3.00] |  |
| ^1^ Edinburgh Postnatal Depression Scale. Number in some variables do not add up to 1070 because of few missing values. | | | | | | |

**Table (5).** Association between antenatal depressive symptoms (defined as EPDS^1^ score≥ 14) and laboratory factors in univariable analysis.

| Characteristics | Total | Prevalence | | Crude Odds Ratio [95% CI] | | p |
| --- | --- | --- | --- | --- | --- | --- |
|  |  | n | (%) |  |  |  |
| Iron (umol/L) |  |  |  |  |  |  |
| <9 umol/L | 325 | 65 | (17.23) |  | [Ref.] | 0.924 |
| 9-30 umol/L (normal) | 708 | 128 | (18.08) | 1.06 | [0.75-1.50] |  |
| > 30 umol/L | 31 | 6 | (19.35) | 1.15 | [0.45-2.94] |  |
|  |  |  |  |  |  |  |
| Ferritin ug/L |  |  |  |  |  |  |
| < 13 ug/L | 340 | 64 | (18.82) |  | [Ref.] | 0.717 |
| 13-150 ug/L (normal) | 683 | 117 | (17.13) | 0.89 | [0.64-1.25] |  |
| >150 ug/L | 44 | 9 | (20.45) | 1.11 | [0.51-2.42] |  |
| Anemia |  |  |  |  |  |  |
| No | 768 | 134 | (17.45) |  | [Ref.] | 0.583 |
| Yes | 302 | 57 | (18.87) | 1.10 | [0.78-1.55] |  |
| Vitamin B_12_ (pg/L) |  |  |  |  |  |  |
| ≥ 180 pg/ml | 729 | 119 | (16.32) |  | [Ref.] | 0.094 |
| 130-179 pg/ml | 225 | 51 | (22.67) | 1.50 | [1.04-2.17] |  |
| < 130 pg/ml | 113 | 20 | (17.70) | 1.10 | [0.65-1.86] |  |
| Calcium |  |  |  |  |  |  |
| >5.52 mmol/L | 113 | 18 | (15.93) | 0.60 | [0.07-4.88] | 0.801 |
| 2.2 - 5.52 mmol/L | 955 | 173 | (18.12) |  | [Ref.] |  |
| <2.2 mmol/L | 955 | 173 | (18.12) | 1.07 | [0.77-1.49] |  |
| RBC Folate ng/mL |  |  |  |  |  |  |
| < 263 ng/mL | 113 | 18 | (15.93) |  | [Ref.] | 0.566 |
| ≥ 263 ng/mL | 955 | 173 | (18.12) | 1.17 | [0.69-1.98] |  |
| Vitamin D status |  |  |  |  |  |  |
| Deficiency/insufficiency (25OHD <75 nmol/L) | 817 | 152 | (18.60) |  | [Ref.] | 0.218 |
| Sufficiency (25OHD ≥ 75 nmol/L) | 250 | 38 | (15.20) | 0.78 | [0.53-1.15] |  |
| ^1^ Edinburgh Postnatal Depression Scale. 25OHD: 25-Hydroxyvitamin D. Number in some variables do not add up to 1070 because of few missing values. | | | | | | |

**Table (6).** Factors associated with antenatal depressive symptoms (defined as EPDS^1^ score≥ 14) in Kuwait birth cohort study in the adjusted model.

| Characteristics | Total | Prevalence | | Adjusted Odds Ratio [95% CI] | | p |
| --- | --- | --- | --- | --- | --- | --- |
|  |  | n | (%) |  |  |  |
| Group (1): Stressors^2^ |  |  |  |  |  |  |
| Wanted to get pregnant |  |  |  |  |  |  |
| No | 331 | 76 | (22.96) |  | [Ref.] | 0.026 |
| Yes | 733 | 115 | (15.69) | 0.67 | [0.48-0.95] |  |
| Husband married to another woman |  |  |  |  |  |  |
| No | 1041 | 181 | (17.39) |  | [Ref.] | 0.036 |
| Yes | 26 | 10 | (38.46) | 2.63 | [1.06-5.97] |  |
| Stressful life event |  |  |  |  |  |  |
| No | 862 | 136 | (15.78) |  | [Ref.] | 0.003 |
| Yes | 196 | 55 | (28.06) | 1.76 | [1.21-2.58] |  |
| Group (2): Mediators^3^ |  |  |  |  |  |  |
| Current use of supplements/vitamins |  |  |  |  |  |  |
| No | 60 | 22 | (36.67) |  | [Ref.] | 0.001 |
| Yes | 999 | 169 | (16.92) | 0.33 | [0.17-0.64] |  |
| Eating meat during the last 3 months |  |  |  |  |  |  |
| I eat both meat and fish | 821 | 138 | (16.81) |  | [Ref.] | 0.004 |
| I avoid meat, but eat fish | 69 | 22 | (31.88) | 2.45 | [1.34-4.48] |  |
| I avoid fish, but eat meat | 103 | 17 | (16.50) | 0.71 | [0.38-1.34] |  |
| I avoid both fish and meat | 49 | 9 | (18.37) | 0.98 | [0.40-2.42] |  |
| I am vegetarian | 12 | 5 | (41.67) | 5.37 | [1.41-20.39] |  |
| Consumption of fresh vegetables (days per week) |  |  |  |  |  |  |
| ≤ 2 days a week | 309 | 100 | (32.36) |  | [Ref.] | 0.015 |
| 3-5 days a week | 279 | 46 | (16.49) | 0.52 | [0.31-0.86] |  |
| ≥6 days a week | 452 | 76 | (16.81) | 0.53 | [0.33-0.86] |  |
| Physical activity (total MET) |  |  |  |  |  |  |
| Low | 354 | 52 | (14.69) |  | [Ref.] | 0.002 |
| Middle | 357 | 69 | (19.33) | 1.28 | [0.80-2.04] |  |
| High | 355 | 103 | (29.01) | 2.15 | [1.38-3.37] |  |
| Pre-pregnancy BMI categories |  |  |  |  |  |  |
| Underweight | 24 | 3 | (12.50) | 0.61 | [0.17-2.04] | 0.039 |
| Healthy weight | 299 | 43 | (14.38) |  | [Ref.] |  |
| Overweight | 363 | 77 | (21.21) | 1.74 | [1.13-2.71] |  |
| Obesity | 276 | 50 | (18.12) | 1.26 | [0.78-2.03] |  |
| ^1^ Edinburgh Postnatal Depression Scale. ^2^Adjusted for nationality (p=0.148), age group (p=0.289), employment (p=0.655), type of housing (p=0.462), age at first pregnancy (categorized)(p=0.288), passive smoking at home (p=0.087), presence of hypertension (p=0.274), and gestational diabetes (p=0.120). ^3^Adjusted for using consumption of fruits (p=0.070), consumption of energy drinks (p=0.644), vitamin B_12_ (p=0.407). | | | | | | |
